# Supplementary material for: Competition between proton transfer and intermolecular Coulombic decay in water
Source: Nat Commun. 2018 Nov 26;9:4988. doi: 10.1038/s41467-018-07501-6 (PMC6255891; doi:10.1038/s41467-018-07501-6)
Supplement: Supplementary file 1 — Supplementary Information [file 41467_2018_7501_MOESM1_ESM.pdf]

Supplementary Information for: Competition between proton transfer and intermolecular Coulombic decay in water

Clemens Richter *et al.*

E-mail:

Supplementary Figures

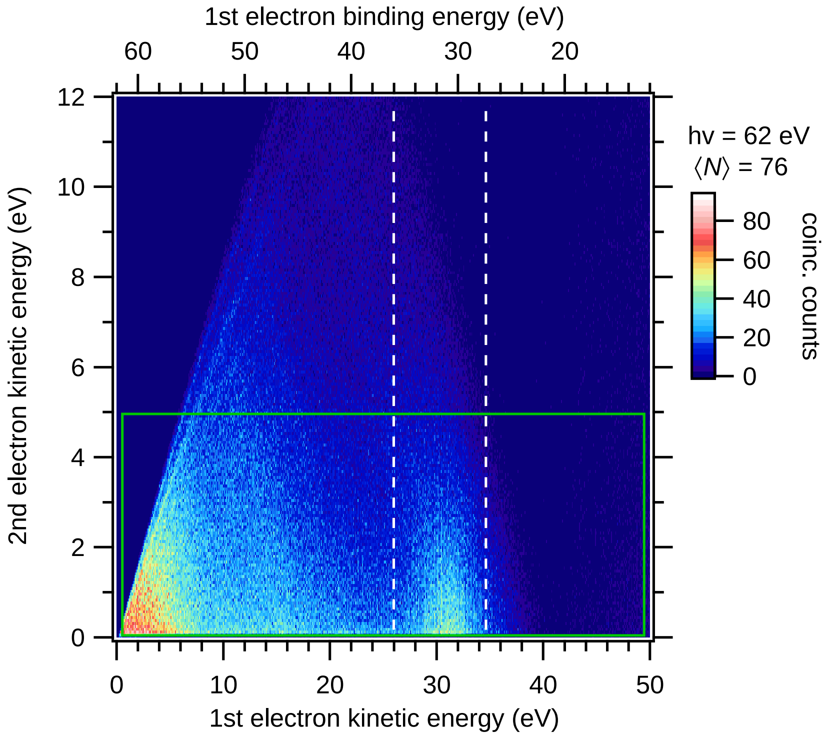

Supplementary Figure 1: Electron-electron coincidence data
Electron-electron coincidence data recorded from a jet of $\langle N \rangle = 76$ water clusters at $h\nu = 62\,\mathrm{eV}$. Intensity is shown as events/pixel of $92 \times 67\,\mathrm{meV}^2$ width, as a function of kinetic energy E_1 of the faster and kinetic energy E_2 of the slower electron. A linear color scale is used. The E_1 interval marked by two vertical bars is assigned to events initiated by $2a_1$ photoionization. The E_2 interval marked by the green box was used to obtain the coincident electron spectra, which were used to determine the ICD efficiency. The total number of coincident events acquired for each cluster size and photon energy was roughly between 4×10^5 and 2×10^6, within an acquisition time of 140-900 s. Further discussion of this type of data in general can be found in Supplementary Ref. 1, and in particular to water in Supplementary Ref. 2.

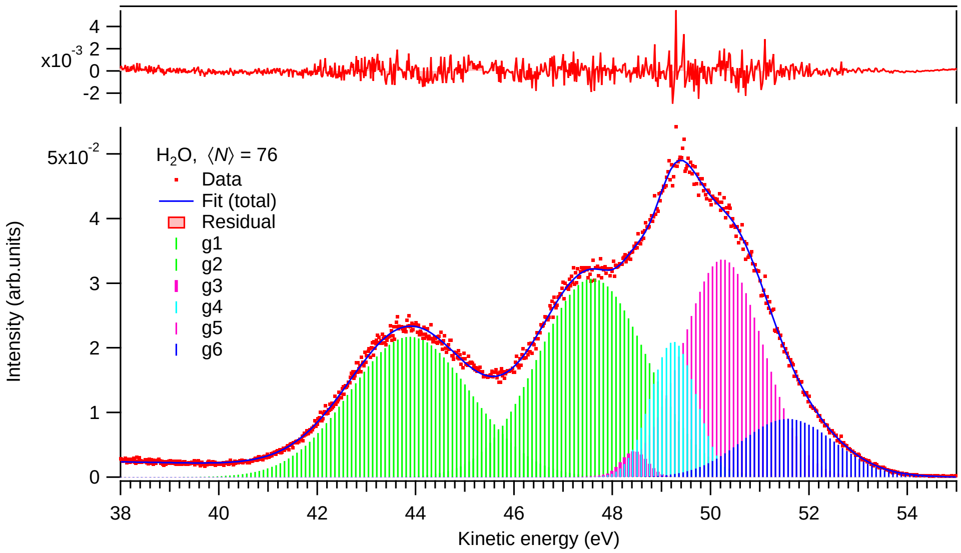

Supplementary Figure 2: Outer valence spectrum used for estimating the degree of condensation

Outer valence photoelectron spectrum of a jet of $\langle N \rangle = 76$ water clusters at $h\nu = 62$ eV. Components of a least squares fit to disentangle cluster and monomer intensity, and the residual of the fit (top trace) are also shown. The components 'g5' and 'g6' are assigned to the cluster HOMO component, components 'g3' and 'g4' are assigned to the monomer $1b_1$, 'g3' tentatively to the $v' = 2$ vibrational component. A number of constraints were applied to produce consistent and well-defined fit results over the whole series of cluster sizes.

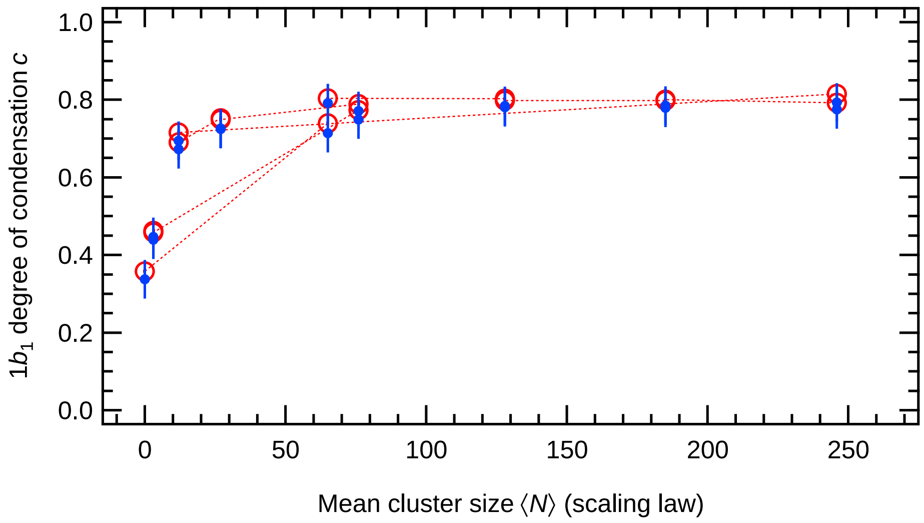

Supplementary Figure 3: Degree of condensation
Degree of condensation c determined from outer valence spectra at $h\nu = 62$ eV (see Supplementary Fig. 2) as a function of mean cluster size $\langle N \rangle$ (see Supplementary Table 1). Small round symbols are from spectra recorded before and after an ICD efficiency measurement; in some cases the two data points are overlapping. An arithmetrical average of the two data points at equal $\langle N \rangle$ was used as an estimate for c. Error bars show the standard deviation. Additionally, larger round symbols show the result of neglecting the small auxiliary peak 'g3' when calculating c, obviously the effect is small. See Supplementary Methods for details.

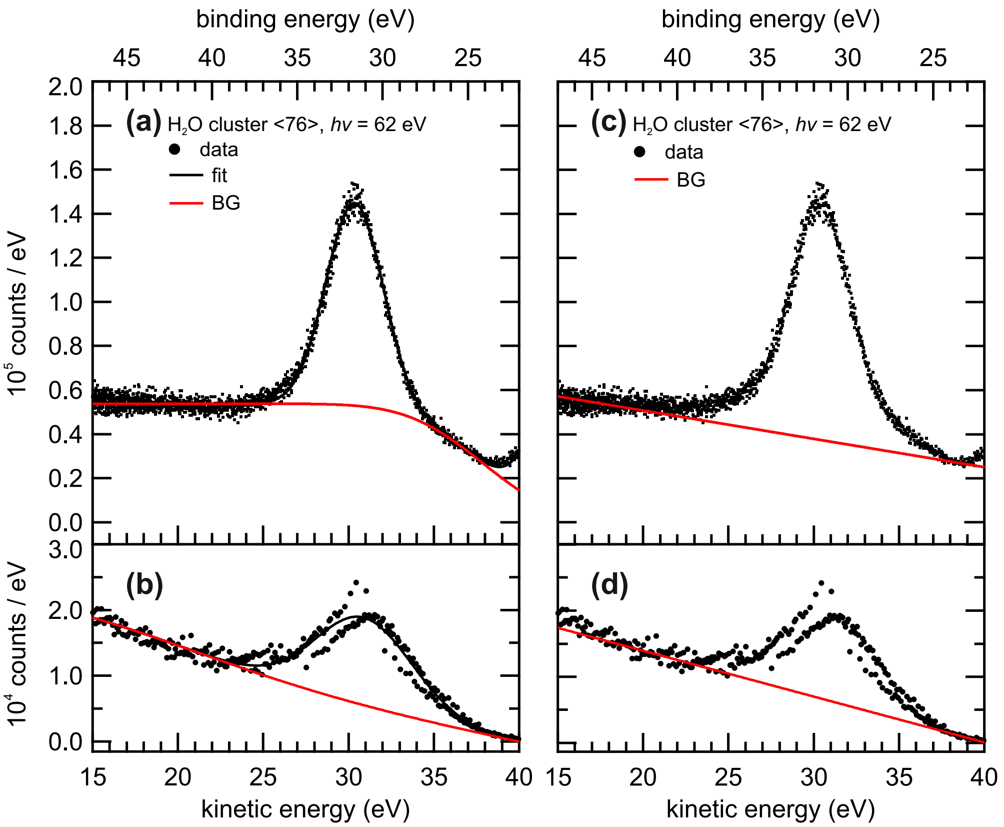

Supplementary Figure 4: Peak-to-background separation in the experimental data used to determine the efficiency of ICD

Coincident and non-coincident electron spectra recorded after photoionization of $\langle N \rangle = 76$ water clusters at $h\nu = 62$ eV: Panels (a) and (c) show $p(E_{\rm ph})$, the intensity of photoelectrons detected in the region of $2a_1$ binding energies; panels (b) and (d) show the integral of $P(E_{\rm ph}, E_{\rm ICD})$ over an interval of $[0, 5]$ eV for $E_{\rm ICD}$ (region marked by a green rectangle in Supplementary Fig. 1). In other words, the latter panels show the intensity of photoelectrons detected in coincidence with an ICD electron. Experimental data in the left and right hand side panels are identical, but different choices for peak-to-background separation are indicated. The black solid traces correspond to the fit of the photoline with background and are shown to guide the eye. The red solid traces in (c) and (d) indicate a linear background below the $2a_1$ peak, while panels (a) and (b) show a curved, Shirley-type background. We believe these two variants mark the largest and smallest choices for the ratio $P(E_{\rm ph}, E_{\rm ICD})/p(E_{\rm ph})$ that are compatible with our data.

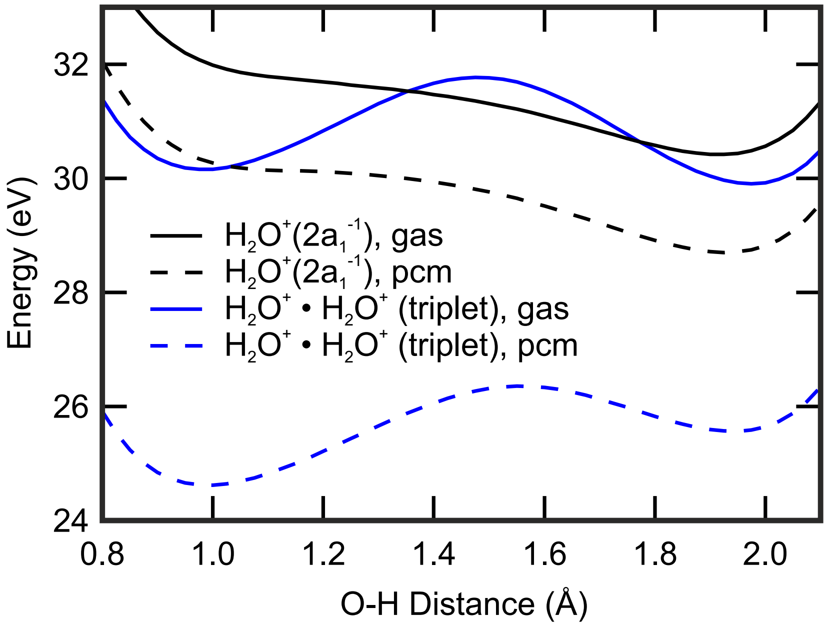

Supplementary Figure 5: Calculated potential curves for proton transfer in a water dimer embedded in a polarizable continuum

Calculations are shown for a gas phase dimer (solid lines) and for the same dimer embedded in a polarizable continuum (dashed lines) and were performed at the PBE0/6-311++G** level. The blue lines depict the lowest doubly ionized triplet state, while the black lines depict the $2a_1$ singly ionized state. See Supplementary Notes 4 for details.

Supplementary Tables

Supplementary Table 1: Parameters of cluster source and calculated mean cluster size $\langle N \rangle$

Expansion conditions for cluster production, and calculated mean cluster size $\langle N \rangle$. All experiments used a conical nozzle with 80 μm smallest diameter and a 15° half opening angle. The stagnation pressure p was derived from the vapour pressure of water at the reservoir temperature T_r. The nozzle temperature T_n was set independently from T_r. The H_2O/D_2O comparison was measured in a separate beamtime. See Supplementary Methods for a detailed description.

Species	T_r (°C)	p (mbar)	T_n (°C)	$\langle N \rangle$	$\langle N \rangle^{-1/3}$
H_2O	76.6	413	144	5.2	0.576
	87.5	637	144	11.8	0.439
	99.5	994	144	27.4	0.332
	114	1630	144	67.1	0.246
	115	1690	144	74.6	0.238
	123	2210	144	123	0.201
	131	2740	144	186	0.175
	135	3150	144	241	0.161
H_2O	104	1180	102	74.7	0.237
H_2O	102	1085	102	63.7	0.250
H_2O	104	1180	104	71.6	0.241
D_2O	102	1080	102	63.7	0.250
D_2O	104	1180	104	71.7	0.241
D_2O	107	1280	104	83.8	0.229

Supplementary Table 2: Single and double ionization potentials of water dimer
Calculated single (SIP) and double (DIP) ionization potentials of $(H_2O)_2$ using different *ab initio* methods. All calculations utilized the aug-cc-pVTZ basis set. The geometry was optimized at the MP2/6-31++G** level. See Supplementary Notes 2 for a detailed description.

	HF	CASSCF	CASPT2	MP2	MP4	CCSD	CCSD(T)	CCSD-DIP
SIP-$2a_1$	32.94	32.71	32.12	32.79	32.10			
DIP-S_0	34.99	26.89	30.08	30.57	25.39	31.46	28.39	30.33
DIP-S_1		29.10	32.14					32.72
DIP-S_2		29.38	32.42					36.36
DIP-T_0	26.61	26.89	(30.16)	30.43	30.16	29.84	30.05	30.32
DIP-T_1		29.10	32.22					32.43
DIP-T_2		29.37	32.48					32.70

Supplementary Table 3: Lifetime calculations
ICD lifetime in fs for the water pentamer estimated from the decay widths of the donor and acceptor subunits of the water dimer, computed with the Fano-CI method. $\alpha_{\mathrm{H_2O}}$ and $\alpha_{\mathrm{D_2O}}$ are the respective ICD efficiencies estimated from equation (4) of the main text, using the values of 2.9 fs and 4.0 fs for t_{open} for the water dimer and the deuterated dimer, respectively. See Supplementary Notes 3 for a detailed description.

	$\hbar/(2\Gamma_a + 2\Gamma_d)$	$\alpha_{\mathrm{H_2O}}$	$\alpha_{\mathrm{D_2O}}$
S+T	21.5	0.13	0.17
2S+2T	7.0	0.34	0.44
3S+3T	5.4	0.42	0.52
all open channels	3.9	0.52	0.64

Supplementary Notes

Supplementary Note 1. Representation of experimental errors

While some of the experimental errors discussed above are stochastic, others influence all data points in one or the other direction ('scale error'). The biggest single source of error is the non-uniqueness in peak-background separation as shown in Supplementary Fig. 4. This leads to a lower and upper bound for α_{ICD}, which is represented by showing two different datasets in Fig. 1c of the main article. The stochastic errors (error in the degree of condensation c, statistical errors) are shown by error bars to these symbols. The remaining systematic errors (error in the mean cluster size $\langle N \rangle$, error in the detection efficiency γ, a 5% relative error introduced to model the neglect of the last factor in supplementary equation (7)) are visualized by the shaded area in Fig. 1c, which shows the effect of these three factors applied to the two dotted lines in the figure.

Supplementary Note 2. Benchmarking the energetics of water dimer

Supplementary Table 2 summarizes the *ab initio* data for the energetics of $2a_1$ singly ionized states, and for doubly ionized states of the water dimer using various electronic structure methods. The same basis set, aug-cc-pVTZ, was utilized throughout.

Let us first focus on the inner valence ionized $2a_1$ state. We are interested in the state in which the hole is located at the donor water molecule, since this type of ionization triggers the proton transfer. To calculate this state using standard single-reference methods, we used the Maximum Overlap Approach[3]. Our best reference value was obtained at the MP4/aug-cc-pVTZ level, which predicts the ionization potential at 32.1 eV, which is about 0.7 eV below the MP2 value. Unfortunately, the MOM calculation at the CCSD and CCSD(T) suffered from convergence difficulties.

We can, however, compare the results with the multireference CASPT2 approach. It

should be noted that it is not immediately clear how to calculate ionization energies using methods based on active space, since the size of the active space will be generally different for states with different number of electrons. In this case, the $2a_1$ ionized state is calculated as an excited singly ionized state using the limited active space of 15 electrons in 8 orbitals. In this active space, the $2a_1$ ionized state is the 7th state, which is calculated to lie 20.4 eV above the first ionized state. If we add the reference HOMO ionization energy (11.73 eV calculated at the MP4 level), we obtain the value which is very close to the MOM-MP4 result.

As expected, the single reference methods behave erratically for the singlet doubly ionized state, since they cannot properly describe its biradical character. On the other hand, active space methods and DIP-EOM-CCSD correctly predict that triplet and singlet ground state DIP have almost the same energy. For the triplet doubly ionized state, the CCSD(T) and MP4 values are very similar, which indicates some robustness of this result (this is not the case for the singlet state). We can take the MP4 DIP triplet as a reference value and look at the excited states using the CASPT2 method, using an active space of 4 electrons in 6 orbitals. The first two singlet and triplet excited states lie only slightly above the inner valence ionized state. Thus, we cannot rule out that they are available as final ICD states near the equilibrium geometry.

Supplementary Note 3. Dependence of ICD lifetime on number of open channels

We discuss here how the number of available final states influences the ICD rate. In our Fano-CI approach, the relative energies of the $2a_1$ inner valence ionized state and the doubly ionized states are similar to those obtained at the CASSCF level given in Supplementary Table 2. Three channels in each spin symmetry are open in the case of the donor, and four channels are open in the case of the acceptor. We computed the ICD lifetimes for each of these channels. The results for the ICD lifetime as a function of the number of channels

included are shown in Supplementary Table 3. Despite the fairly large changes in the ICD lifetime depending on the number of allowed channels, the predicted efficiency (10-45%) agrees well with the experiments (5-20%) for the smaller clusters considered here.

Supplementary Note 4. Potential energy curves in polarizable continuum model

To estimate the energetics of the ICD process in liquid water, we performed a scan along the proton transfer coordinate using water dimer embedded in a dielectric continuum as a model. The technical details of these calculations are described below. The results are shown in Supplementary Fig. 5.

The curves for the both initial and final ICD states are qualitatively similar to the gas phase, but both curves are shifted to lower energies as the solvent stabilizes the extra positive charge. This stabilization is more pronounced for the doubly ionized final state, resulting in the ICD electrons with higher kinetic energies. In fact, our results suggest that the ICD channel never closes in liquid water. Note that this is a rather crude model and experimental verification is needed for this conjecture. Nevertheless, the widening of the gap between initial and final state is clearly apparent in the measured data as shown in Fig. 6 of the main article.

Let us now comment on the technical aspects of these calculations. Here, we focused only on the initial singly ionized $2a_1^{-1}$ state and the lowest triplet doubly ionized state, which can both be reasonably described by single reference methods as shown in Table 2. We used the PBE0 functional with the 6-311++G** basis set, but the gas phase energies were shifted to match the reference MP4 values. The same approach was used for Fig. 3 in the article. There are two aspects of these calculations that deserve further discussion: 1. the use of single reference DFT method to obtain the energies of highly excited single ionized state and 2. the choice of the polarizable continuum model.

As explained in the Methods section, highly excited states can be obtained with the help

of the Maximum Overlap Method (MOM)[3,4]. However, straightforward use of the MOM leads to energy discontinuities along the proton transfer coordinate. We have also tested a recently published Improved MOM (IMOM) method[5], but with no improvement for this problem. In the end, we were able to make use of the fact that the inner valence orbital of the donor water molecule is energetically well separated from the other orbitals. Therefore, instead of using the standard MOM criterion, we simply populated the desired orbitals based on the energy criterion. We implemented this approach in the development version of the TeraChem package for both MOM and IMOM methods[6,7].

To model the solvent effects, we used the non-equilibrium formulation of the C-PCM model as implemented in the TeraChem package[8]. Although, the non-equilibrium solvation takes into account that the ionization is a vertical process, the overall ICD process that we are trying to describe here is not vertical and the surrounding water molecules have certain (albeit short) time to relax around the ionized molecule. The dynamical aspect of this process is hard to describe by the PCM methodology, but these effects would not changes the qualitative conclusions.

Supplementary Methods

Cluster production and size estimation: In our work, water clusters were formed via a supersonic expansion of pure water vapour into vacuum (no seeding gas was used). The jet produced such contains clusters with a broad distribution of sizes N, including monomers. The mean of this distribution $\langle N \rangle$ can be estimated from the parameters of the expansion. For this estimate, we use an empirical relation due to Bobbert *et al.*[9] Details, and a critical account of this approach to cluster size estimation, have been given by some of the authors (Supplementary ref. 10, see 'Supporting Information' of the cited work). In Supplementary Table 1 we give the expansion parameters, and calculated mean cluster sizes. Following Supplementary ref. 10, we assign to those a systematic uncertainty of 35%, including a 7% uncertainty in the reference temperature of the thermocouple used to measure the reservoir temperature, and the remainder attributed to limitations inherent in the model. We expect these factors, if present, to be common to all data points (all sizes given are consistently too small or too large).

Expression for the ICD efficiency: In the following, we give some details on the derivation of the expression that connects the experimental data with the ICD efficiency. Following that, we analyze the errors assigned to its individual factors.

The process we consider starts by photoionization, creating a photoelectron of kinetic energy E_{ph}. After that, the target is left in an excited state and emits another electron (the ICD electron) with kinetic energy E_{ICD}. By tuning the photon energy we can always achieve $E_{\text{ph}} \neq E_{\text{ICD}}$, *i.e.* photoelectron and secondary electron can be distinguished experimentally. In an ideal experiment, the branching ratio α_{ICD} of the autoionization process ($\alpha_{\text{ICD}} \in [0, 1]$) can then be derived from experimentally measurable data as:

$$\alpha_{\text{ICD}} = \frac{P(E_{\text{ph}}, E_{\text{ICD}})}{p(E_{\text{ph}})}, \qquad (1)$$

where P is the rate for detection of electron pairs with energies E_{ph} and E_{ICD}, and p the rate

for detection of photoelectrons with energy $E_{\rm ph}$ (with or without a subsequent secondary electron). Rigorously, both photoelectrons and autoionization electrons, are emitted within some intervals of kinetic energy. In the following, $E_{\rm ph}$ and $E_{\rm ICD}$ rather designate the central values of the respective intervals $\overline{E_{\rm ph}}, \overline{E_{\rm ICD}}$, and $P(E_{\rm ph}, E_{\rm ICD}) \equiv P(\overline{E_{\rm ph}}, \overline{E_{\rm ICD}})$, $p(E_{\rm ph}) \equiv p(\overline{E_{\rm ph}})$. In our experiments, we always had $E_{\rm ph} > E_{\rm ICD}$, therefore we designate the photo- and ICD electron the 'first' and 'second' electron to arrive at the detector, with kinetic energies E_1 and E_2.

In our actual experiment, several corrections of the measured data must be taken into account, leading to a more complicated version of supplementary equation (1). These are:

1. the less-than-unity detection efficiency of the spectrometer,
2. the background of uncondensed monomers in the cluster jet,
3. losses by intracluster inelastic electron scattering,
4. a difference in the outer valence photoionization cross section of monomers and clusters.

The first two points lead to a correction factor of $\big(c\gamma(E_{\rm ICD})\big)^{-1}$ in the expression for $\alpha_{\rm ICD}$. Here, c is the degree of condensation (ratio of molecules being a part of a cluster to total number of molecules in the interaction region) and $\gamma(E_{\rm ICD})$ the detection efficiency of the spectrometer for ICD electrons. The detection efficiency for photoelectrons $\gamma(E_{\rm ph})$ cancels from the expression.

Further complications occur due to the overlap of the inner valence monomer and cluster photoelectron lines for molecular clusters. We consider the observed rate of photoelectrons $p(E_{\rm ph}) = \gamma(E_{\rm ph}) r_{ph}$. It contains a rate $p(E_{\rm ph}) > \tilde{p}(E_{\rm ph}) = \gamma(E_{\rm ph}) r_{ph} c$ from clusters, which is not directly observable due to the overlap. We would also like to take into account the effect of inelastic intracluster photoelectron scattering, which diminishes $\tilde{p}(E_{\rm ph})$. (Densities in our jet are such that inelastic scattering at other molecules or clusters can be neglected.) We describe the inelastic losses by a factor $f := 1-(\textit{lost fraction})$. With that, the observed

rate of photoelectrons becomes

$$p(E_{\mathrm{ph}}) = \gamma(E_{\mathrm{ph}}) r_{\mathrm{ph}} \big[(1-c) + cf\big], \tag{2}$$

because the correction by f must only be applied to the cluster part of the intensity. We assume that no correction for intercluster scattering is needed for the ICD electrons, because their energy is low and low cross sections for inelastic electron scattering in amorphous ice were found at these energies[11,12]. For the ratio with the coincident events we now have

$$\frac{P(E_{\mathrm{ph}}, E_{\mathrm{ICD}})}{p(E_{\mathrm{ph}})} = \frac{\gamma(E_{\mathrm{ph}}) r_{\mathrm{ph}}\, cf\, \gamma(E_{\mathrm{ICD}}) \alpha_{\mathrm{ICD}}}{\gamma(E_{\mathrm{ph}}) r_{\mathrm{ph}} \big[(1-c) + cf\big]} \quad \Leftrightarrow \quad \alpha_{\mathrm{ICD}} = \frac{P(E_{\mathrm{ph}}, E_{\mathrm{ICD}})}{p(E_{\mathrm{ph}})} \frac{1}{c\, \gamma(E_{\mathrm{ICD}})} \left(\frac{1-c}{f} + c \right). \tag{3}$$

The degree of condensation c in our experiments is determined by electron spectroscopy as well, and results from a comparison of the features from condensed molecules vs. monomers in a region of the spectrum were they can be distinguished. Practically, the HOMO levels of molecules and clusters were compared (see below). We use:

$$c = \frac{r_{\mathrm{c}}}{r_{\mathrm{c}} + r_{\mathrm{m}}}, \tag{4}$$

where c and m refer to clusters and monomers, respectively. This implicitly supposes that the measured photoelectron intensities are proportional to the respective numbers of condensed and uncondensed molecules in the interaction region, which is the case if their outer valence photoionization cross sections are equal—an assumption often made in PE studies of clusters, which, however, has rarely been checked rigorously[13]. We would like to allow for a correction factor taking into account possible differences between the water HOMO cross section for the monomer (σ_{m}, the $1b_1$ cross section) and for a single water molecule within a cluster, σ_{c}. Defining $x := \sigma_{\mathrm{m}}/\sigma_{\mathrm{c}}$ we can write a cross-section corrected degree of condensation as

$$\tilde{c} = \frac{x r_{\mathrm{c}}}{x r_{\mathrm{c}} + r_{\mathrm{m}}}. \tag{5}$$

The expected case is $\sigma_c < \sigma_m$, because the orbitals are more diffuse in a cluster, and because in larger clusters molecules at the side of the cluster that is exposed to the photon beam may shadow others. In this case $x > 1$, and the actual degree of condensation $\tilde{c}$ is larger than the calculation of c given in supplementary equation (4). The connection to the measured c is given by

$$\tilde{c} = c\,\frac{x}{cx + 1 - c}. \tag{6}$$

Using $\tilde{c}$ instead of c in supplementary equation (3) after some algebra yields our final result

$$\alpha_{\mathrm{ICD}} = \frac{P(E_{\mathrm{ph}}, E_{\mathrm{ICD}})}{p(E_{\mathrm{ph}})}\,\frac{1}{c\,\gamma(E_{\mathrm{ICD}})}\left(\frac{1-c}{fx} + c\right). \tag{7}$$

We would like to note that most likely f is a number slightly smaller than one, while x should be slightly larger than one, as argued above. In this case, the approximate neglect of the last factor in supplementary equation (7) seems justified. This approximation was used in earlier work on rare gas clusters by some of the authors[14,15] and will again be used here. Additional justification arising from details of our experimental procedures is given below.

In the following, we describe how the individual factors making up supplementary equation (7) were determined.

Spectrometer efficiency: Experiments were carried out with a magnetic bottle time-of-flight spectrometer for electrons[16]. The transmission function $\gamma(E)$ of this instrument is the product of accepted solid angle (as a fraction of 4π) times probability to register a charged particle on the detector, and may weakly depend on kinetic energy E. In supplementary equation (7), we need the value of γ for the kinetic energy interval in which ICD may occur. We determined this property of the spectrometer from measurements of Xe NOO photoelectron-Auger electron coincidences, as described in Supplementary ref. 16. Values for kinetic energies of approx. 0.5 to 5.5 eV were found inbetween 0.5 and 0.6, with no significant energy dependence. In the following, we use their average of $\gamma(E_{\mathrm{ICD}}) = 0.58(4)$.

For the data in the comparison of normal to deuterated water, a value of $\gamma(E_{\mathrm{ICD}}) = 0.39$

was used. This is however less well bounded than the one for the pure water data. The significance of the normal to deuterated water comparison however is not influenced by that.

Degree of condensation: In supplementary equation (7), a correction of the degree of condensation c in the cluster jet is applied, because the $2a_1$ photolines from monomers and clusters cannot be separated. To experimentally determine c, we measured the outer valence photoelectron spectrum of our cluster jet, as it was shown that cluster and monomer signals for the HOMO can be distinguished.[10] We determined c from the area ratio of the two features. In monomers, this line results from ionization of the lone-pair orbital and is dominated by a sharp $v' = 0$ vibrational component. In clusters, it develops a broad peak at a lower binding energy than the monomer. Outer valence spectra were measured at $h\nu = 62$ eV before and after the spectra used for ICD efficiency determination. A retardation voltage of $V_{ret} = -15$ V was used to improve the energy resolution. Nevertheless, peak fitting had to be used to disentangle the cluster from the monomer features (Supplementary Fig. 2). Results for the degree of condensation c (supplementary equation (4)) are shown in Supplementary Fig. 3. Here, symbols of different color are shown for an analysis using either only the fit component 'g4', or both 'g4' and the small auxiliary component 'g3' as the monomer area. Differences between these two approaches and differences between spectra acquired before and after the ICD efficiency measurement amount to a compound estimated error for c of ± 0.05 (standard deviation).

The comparison of normal to deuterated water was measured in a separate beamtime. Here, a similar approach was used to yield $c = 0.75(3)$ for H_2O and $c = 0.74(3)$ for D_2O clusters, however determined at $h\nu = 30$ eV.

Losses by inelastic scattering; Cluster-monomer cross section differences: Loss of photoelectrons in clusters by inelastic scattering is discussed e.g. in Supplementary ref.s 1,13, and can be substantial.

An estimate could be done similar to Supplementary ref. 17, but using inelastic cross sections instead of the elastic cross sections that were of interest in the cited work. To

give an order of magnitude, $f \sim 0.8$. Here we would like to take a different approach, however. Recalling that $x\sigma_c = \sigma_m$, we observe that inelastic losses will also influence the area of cluster outer valence photoelectron lines used in the determination of the degree of condensation. Therefore, if we ignore the slight dependence of inelastic scattering cross section on kinetic energy, $1/f$ will be one of the components making up x, the other being 'intrinsic' changes in the photoionization cross section due to changes of the orbital shape upon aggregation. The change in orbital shape might be substantial[10,18], but whether that affects the photoionization cross section is currently unknown. In the absence of further information, we think it is a fair approximation to propose cancellation of the product fx, which will yield unity for the whole round bracketed factor in supplementary equation (7). Modelling the possible influence of this factor when relaxing the former approximation, we find that it mostly plays a role when the degree of condensation is low. For $c = 0.8$, a $\pm 20\%$ intrinsic difference in the photoionization cross sections will have less than 5% influence on the result for α_{ICD}.

Extraction of spectral intensities from the experimental spectra: Finally, values of the coincident signal intensity P and non-coincident signal intensity p in supplementary equation (7) were determined from the number of events with electrons registered in some interval of kinetic energies. Typical coincident and non-coincident electron spectra are shown in Supplementary Fig. 4, together with the two background models we have applied.

Supplementary References

(1) Mucke, M., Arion, T., Förstel, M., Lischke, T. & Hergenhahn, U. Competition of inelastic electron scattering and Interatomic Coulombic Decay in Ne clusters. *J. Electron Spectros. Relat. Phenom.* **200**, 232–238 (2015). URL http://www.sciencedirect.com/science/article/pii/S0368204815000894.

(2) Mucke, M. *et al.* A hitherto unrecognized source of low-energy electrons in water. *Nat. Phys.* **6**, 143–146 (2010). URL http://www.nature.com/doifinder/10.1038/nphys1500.

(3) Besley, N. A., Gilbert, A. T. B. & Gill, P. M. W. Self-consistent-field calculations of core excited states. *J. Chem. Phys.* **130**, 124308 (2009). URL http://scitation.aip.org/content/aip/journal/jcp/130/12/10.1063/1.3092928.

(4) Gilbert, A. T. B., Besley, N. A. & Gill, P. M. W. Self-Consistent Field Calculations of Excited States Using the Maximum Overlap Method (MOM). *J. Phys. Chem. A* **112**, 13164–13171 (2008). URL http://pubs.acs.org/doi/abs/10.1021/jp801738f.

(5) Barca, G. M., Gilbert, A. T. & Gill, P. M. Simple Models for Difficult Electronic Excitations. *J. Chem. Theory Comput.* **14**, 1501–1509 (2018).

(6) Ufimtsev, I. S. & Martinez, T. J. Quantum Chemistry on Graphical Processing Units. 3. Analytical Energy Gradients, Geometry Optimization, and First Principles Molecular Dynamics. *J. Chem. Theory Comput.* **5**, 2619–2628 (2009). URL http://pubs.acs.org/doi/abs/10.1021/ct9003004.

(7) Titov, A. V., Ufimtsev, I. S., Luehr, N. & Martínez, T. J. Generating efficient quantum chemistry codes for novel architectures. *J. Chem. Theory Comput.* **9**, 213–221 (2013).

(8) Liu, F., Luehr, N., Kulik, H. J. & Martínez, T. J. Quantum Chemistry for Solvated Molecules on Graphical Processing Units Using Polarizable Continuum Models. *J. Chem. Theory Comput.* **11**, 3131–3144 (2015). URL http://pubs.acs.org/doi/10.1021/acs.jctc.5b00370.

(9) Bobbert, C., Schütte, S., Steinbach, C. & Buck, U. Fragmentation and reliable size distributions of large ammonia and water clusters. *Eur. Phys. J. D* **19**, 183–192 (2002).

(10) Barth, S. *et al.* Valence ionization of water clusters: from isolated molecules to bulk. *J. Phys. Chem. A* **113**, 13519–27 (2009). URL http://www.ncbi.nlm.nih.gov/pubmed/19856943.

(11) Michaud, M., Wen, A. & Sanche, L. Cross sections for low-energy (1100 ev) electron elastic and inelastic scattering in amorphous ice. *Radiat. Res.* **159**, 3–22 (2003). URL https://doi.org/10.1667/0033-7587(2003)159[0003:CSFLEE]2.0.CO;2.

(12) Itikawa, Y. & Mason, N. Cross Sections for Electron Collisions with Water Molecules. *J. Phys. Chem. Ref. Data* **34**, 1–22 (2005). URL http://dx.doi.org/10.1063/1.1799251.

(13) Hergenhahn, U. *et al.* 3p valence photoelectron spectrum of Ar clusters. *Phys. Rev. B* **79**, 155448 (2009). URL http://link.aps.org/doi/10.1103/PhysRevB.79.155448.

(14) Förstel, M., Arion, T. & Hergenhahn, U. Measuring the efficiency of interatomic coulombic decay in Ne clusters. *J. Electron Spectrosc. Relat. Phenom.* **196**, 54–57 (2014). URL http://dx.doi.org/10.1016/j.elspec.2014.04.008.

(15) Förstel, M. *et al.* Long-Range Interatomic Coulombic Decay in ArXe Clusters: Experiment and Theory. *J. Phys. Chem. C* **120**, 22957–22971 (2016). URL http://pubs.acs.org/doi/abs/10.1021/acs.jpcc.6b06665.

(16) Mucke, M. *et al.* Performance of a short "magnetic bottle" electron spectrometer. *Rev. Sci. Instrum.* **83**, 063106 (2012). URL `http://www.ncbi.nlm.nih.gov/pubmed/22755614`.

(17) Zhang, C. *et al.* The photoelectron angular distribution of water clusters. *J. Chem. Phys.* **138**, 234306 (2013). URL `http://link.aip.org/link/JCPSA6/v138/i23/p234306`.

(18) Cabral do Couto, P., Estácio, S. G. & Costa Cabral, B. J. The Kohn-Sham density of states and band gap of water: From small clusters to liquid water. *J. Chem. Phys.* **123**, 54510 (2005). URL `http://link.aip.org/link/?JCP/123/054510/1`.
